# Do patient-specific instruments (PSI) for UKA allow non-expert surgeons to achieve the same saw cut accuracy as expert surgeons?

**DOI:** 10.1007/s00402-018-3031-9

**Published:** 2018-09-03

**Authors:** Gareth G. Jones, K. Logishetty, S. Clarke, R. Collins, M. Jaere, S. Harris, J. P. Cobb

**Affiliations:** MSk Lab, Imperial College London, 7th Floor Lab Block Charing Cross Hospital, London, W6 8RF UK

**Keywords:** Unicompartmental knee arthroplasty, Partial knee replacement, Patient-specific instrumentation, PSI, Patient-specific guides, 3D printing

## Abstract

**Introduction:**

High-volume unicompartmental knee arthroplasty (UKA) surgeons have lower revision rates, in part due to improved intra-operative component alignment. This study set out to determine whether PSI might allow non-expert surgeons to achieve the same level of accuracy as expert surgeons.

**Materials and methods:**

Thirty-four surgical trainees with no prior experience of UKA, and four high-volume UKA surgeons were asked to perform the tibial saw cuts for a medial UKA in a sawbone model using both conventional and patient-specific instrumentation (PSI) with the aim of achieving a specified pre-operative plan. Half the participants in each group started with conventional instrumentation, and half with PSI. CT scans of the 76 cut sawbones were then segmented and reliably orientated in space, before saw cut position in the sagittal, coronal and axial planes was measured, and compared to the pre-operative plan.

**Results:**

The compound error (absolute error in the coronal, sagittal and axial planes combined) for experts using conventional instruments was significantly less than that of the trainees (11.6°±4.0° v 7.7° ±2.3º, *p* = 0.029). PSI improved trainee accuracy to the same level as experts using conventional instruments (compound error 5.5° ±3.4º v 7.7° ±2.3º, *p* = 0.396) and patient-specific instruments (compound error 5.5° ±3.4º v 7.3° ±4.1º, *p* = 0.3). PSI did not improve the accuracy of high-volume surgeons (*p* = 0.3).

**Conclusions:**

In a sawbone model, PSI allowed inexperienced surgeons to achieve more accurate saw cuts, equivalent to expert surgeons, and thus has the potential to reduce revision rates. The next test will be to determine whether these results can be replicated in a clinical trial.

## Introduction

Unicompartmental knee arthroplasty (UKA) is an attractive option in patients with mono-compartment gonarthrosis. Compared to total knee arthroplasty (TKA), it is associated with a lower risk profile, shorter length of stay, restoration of a more physiological gait, and higher outcome scores [1–4]. However, UKA is also associated with higher revision rates, and this may be an important factor in explaining why it accounts for less than 10% of knee replacements in a number of national joint registries [5–7].

Lower revision rates for UKA are consistently observed in high-volume surgeons and specialist centres [8–11]. The aetiology of this caseload effect is likely to be multifactorial. One of these factors is that UKA is a technically demanding procedure with a narrow tolerance for tibial component malpositioning; deviations of more than 3° from the native joint line in the coronal plane and 2° in the sagittal plane are associated with decreased prosthesis survival [12, 13]. In this context, the results of a randomised controlled trial RCT comparing conventional and patient-specific instrumentation for UKA are encouraging [14]. The authors, who are both high-volume UKA surgeons, found no difference in component alignment between the two techniques, suggesting that PSI might be able to replicate expert results [15].

The aim of our sawbone study was to ascertain whether PSI allows inexperienced UKA surgeons to achieve the same level of accuracy as expert surgeons by comparing their ability to achieve a planned medial UKA tibial saw cut in the coronal, sagittal and axial planes.

## Materials and methods

**Fig. 1:**
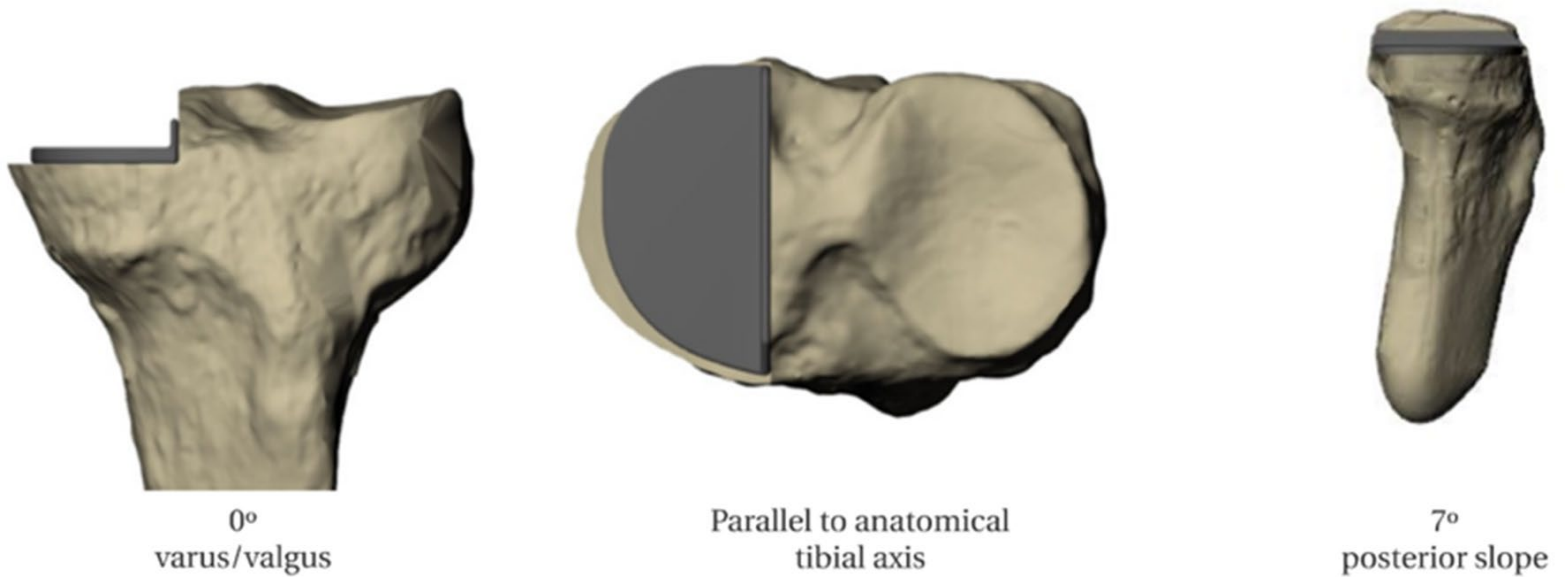
Planned UKA position

Seventy-six identical sawbone tibias, manufactured in the same batch, were used (Sawbones Europe AB, Malmo, Sweden). To create a standard reference, one of these tibias was CT scanned with 1 mm-thick slices and segmented using Mimics software (Materialise NV, Leuven, Belgium) to produce a 3D model. Using 3-matic software (Materialise NV, Leuven, Belgium), this bone model was aligned in 3D space using established frames of reference: the tibial mechanical axis in the Z plane, and the anatomical tibial axis in the *X* and *Y* planes [16]. A virtual tibial bone cut for an Oxford medial compartment UKA (Zimmer Biomet, Bridgend, UK) was then planned 4 mm below the joint line, with a coronal varus/valgus angulation of 0°, a posterior slope of 7° (as delivered by conventional instrumentation when the guide shaft is aligned parallel with the tibial long axis [17]), and an axial orientation parallel to the anatomical tibial axis [16] (Fig. 1).

**Fig. 2:**
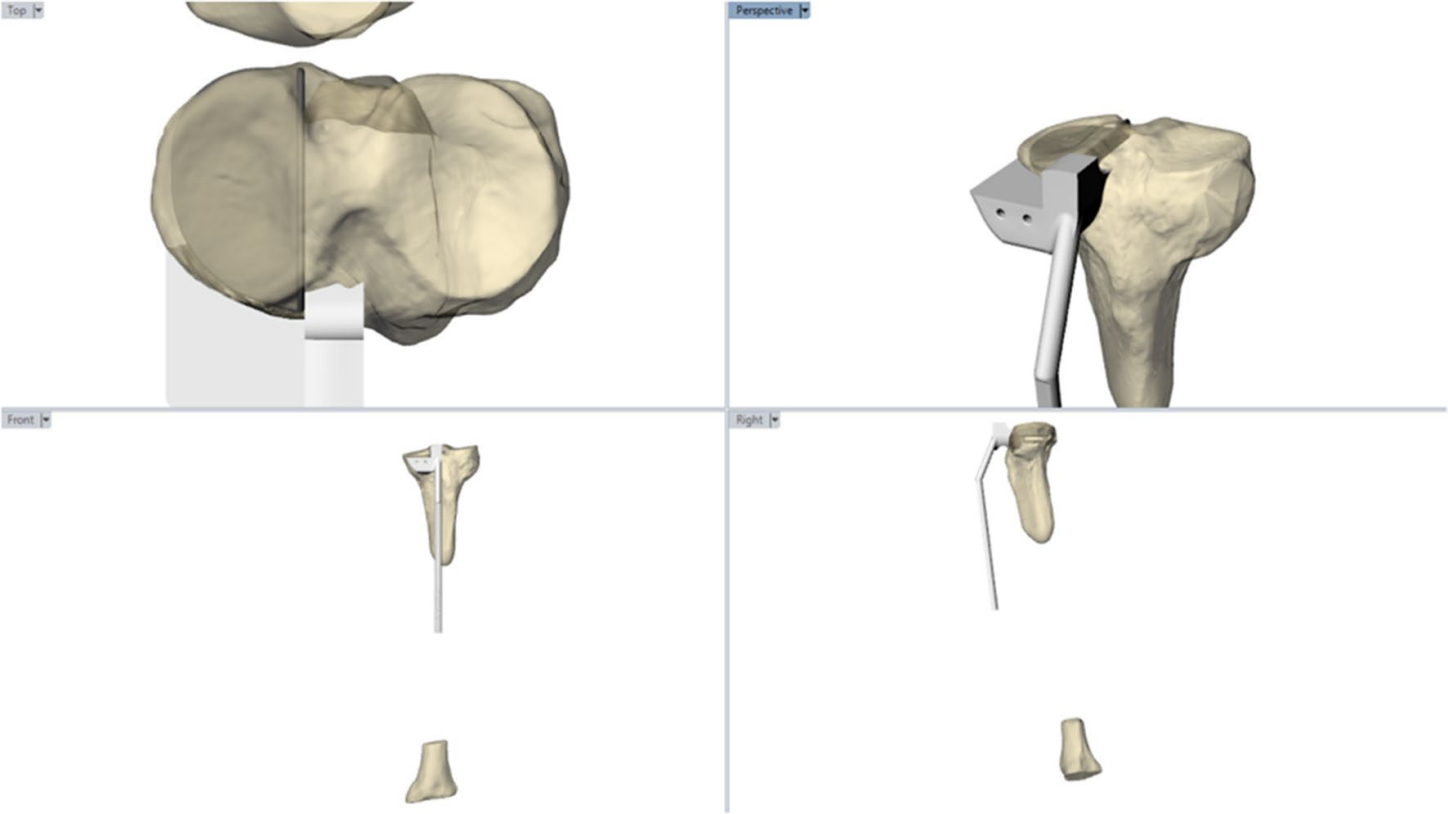
PSI for UKA saw cuts

A PSI to deliver this surgical plan through a standard minimally invasive incision was 3D printed in medical grade Nylon (PA 2200) using an EOS P110 (Embody, London, UK) (Fig. 2). The guide was designed to be used in the same manner as the Oxford Phase III conventional instruments (Zimmer Biomet, Bridgend, UK), and the distal portion of the guide manufactured to fit the conventional ankle clamp.

A minimum sample size of four surgeons in each group was calculated based on a 5% significance level, a power of 80%, a standard deviation of 1.25 estimated from Ollivier et al.’s RCT [14], and a desire to detect a 3° difference in coronal implant orientation [13, 14]. Four expert UKA surgeons (defined as performing more than 30 cases per year [9]) and 34 surgical trainees (no prior experience of UKA surgery) were recruited. All participants watched a video demonstration of the tibial saw cuts for a medial UKA using both conventional Oxford Phase III (Zimmer Biomet, Bridgend, UK) and PSI instruments (Embody, London, UK).

The simulation model used by the implant manufacturer (Zimmer Biomet, Bridgend, UK) for their instructional courses was replicated in this study, i.e. a sawbone knee joint with three stretch tube ligaments mimicking the LCL, MCL and PCL (Model number 1148-1, Sawbones Europe AB, Malmo, Sweden), held in a holder from the same company, that simulated soft tissue and skin around the tibia and femur, whilst allowing free access to the knee joint in a position encountered in the operating theatre (Model 1506). Each participant was provided with printouts of Fig. 1 and asked to achieve this pre-operative plan twice: once with PSI, and once with conventional instruments. Half the participants started first with PSI, and half with the conventional instruments, before swapping over. Oscillating (12 mm × 90 mm) and reciprocating (70 mm × 10 mm) sawblades (De Soutter Medical, Buckinghamshire, UK) designed for clinical use in UKA were used to perform the cuts.

Each cut tibia was CT scanned (1 mm thick slices) and segmented using Mimics software (Materialise NV, Leuven, Belgium) to produce a 3D model. This was surface matched with the pre-operative 3D bone model to ensure identical orientation in space. Acrobot planning software (Acrobot, UK), validated for clinical use, was then used to position a tibial component onto the cut bone surface, and the varus/valgus, posterior slope, and axial rotation recorded. This process was performed by a blinded independent observer and repeated on a random selection of ten bones 1 week later. A second observer (GGJ) repeated the measurements on the same randomly selected ten bones.

SPSS v22 (IBM Corp, New York, USA) was used for statistical analysis. Paired *t* tests were used to compare within group differences, and unpaired *t* tests to compare between groups. A Welch independent *t* test was used if the homogeneity of variances was found to be unequal. Statistical significance was set at *p* < 0.05.

## Results

**Fig. 3:**
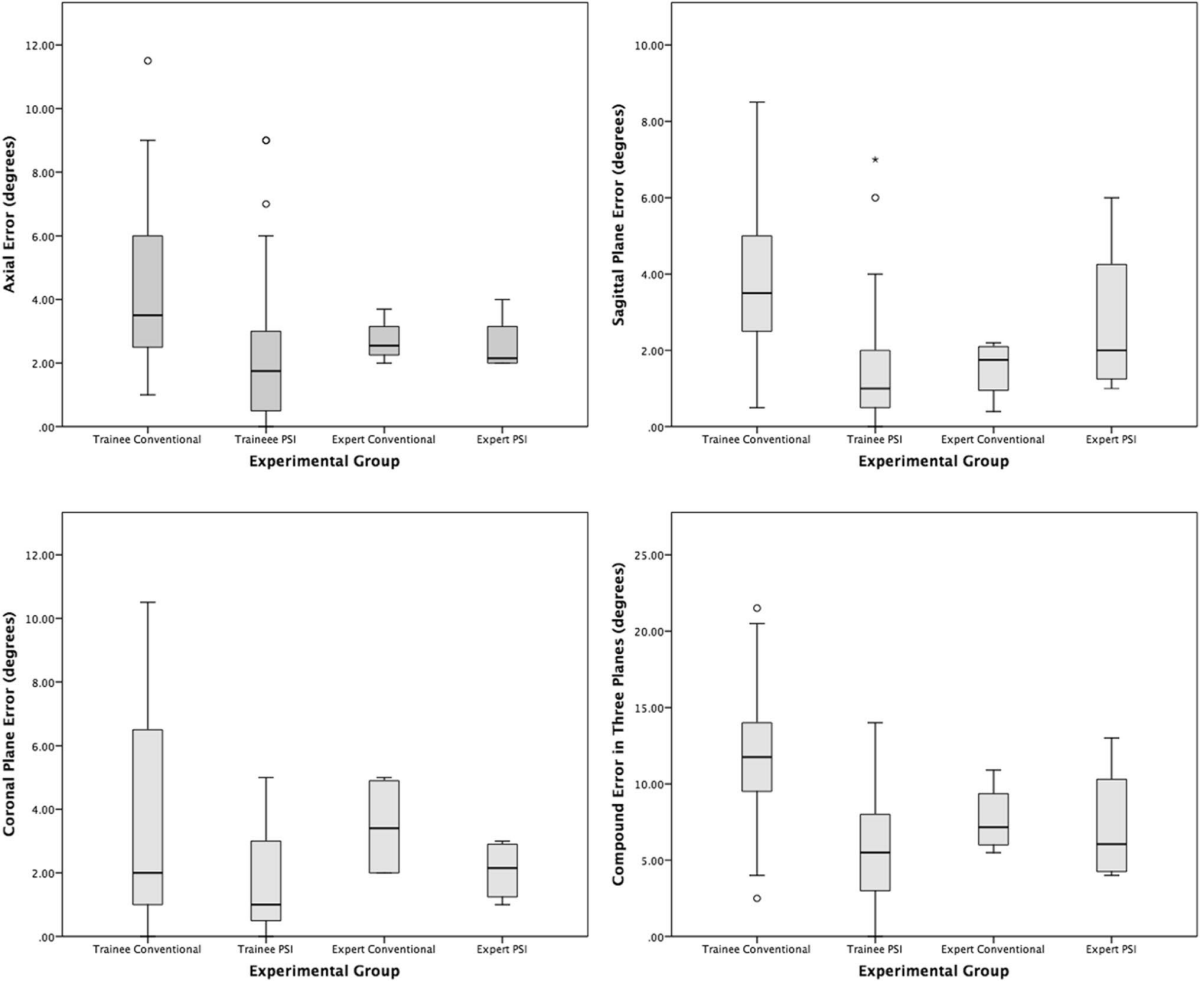
Box plots of the absolute error (degrees) between the planned and achieved implant positions in the coronal, sagittal, and axial planes, plus compound error of all three planes

The absolute difference between the planned and achieved implant position was considered for all analyses, and is referred to simply as the ‘error’. Coronal, sagittal and axial plane measurements were considered separately, and together as a compound error (Fig. 3). The *p* values of differences between the groups are displayed in Tables 1 and 2.

**Table 1:** Comparison of mean absolute error in implant position (SD) between experts and trainees using conventional and patient-specific (PSI) instrumentation

Plane	Trainees	Experts	*p* value	Trainees	Experts	*p* value	Trainees	Experts	*p* value
	Conventional instruments	Conventional instruments		PSI	Conventional instruments		PSI	PSI	
Coronal	3.5° (3.0°)	3.5° (1.7°)	0.951	1.7° (1.6°)	3.5° (1.7°)	0.045	1.7° (1.6°)	1.7° (1.6°)	0.633
Sagittal	3.8° (2.0°)	1.5° (0.8°)	0.039	1.7° (1.7°)	1.5° (0.8°)	0.863	1.7° (1.7°)	2.8° (2.3°)	0.254
Axial	4.4° (2.5°)	2.7° (0.7°)	0.009	2.2° (2.4°)	2.7° (0.7°)	0.713	2.2° (2.4°)	2.6° (1.0°)	0.789
Compound	11.6° (4.0°)	7.7° (2.3°)	0.029	5.5° (3.4°)	7.7° (2.3°)	0.396	5.5° (3.4°)	7.3° (4.1°)	0.3

**Table 2:** Comparison of mean absolute error in implant position (SD) using conventional and patient-specific (PSI) instrumentation for trainees and experts

Plane	Trainees		*p* value	Experts		*p* value
	Conventional	PSI		Conventional	PSI	
Coronal	3.5° (3.0°)	1.7° (1.6°)	0.001	3.5° (1.7°)	1.7° (1.6°)	0.037
Sagittal	3.8° (2.0°)	1.7° (1.7°)	< 0.0005	1.5° (0.8°)	2.8° (2.3°)	0.470
Axial	4.4° (2.5°)	2.2° (2.4°)	0.002	2.7° (0.7°)	2.6° (1.0°)	0.845
Compound	11.6° (4.0°)	5.5° (3.4°)	< 0.0005	7.7° (2.3°)	7.3° (4.1°)	0.849

### Conventional Instruments

For both groups using conventional instruments, the experts’ mean error was significantly lower than the trainees’ in both the axial and sagittal planes, but interestingly not in the coronal plane (Table 1). The overall compound error was significantly lower for the expert group.

#### PSI

Compared to conventional instruments, PSI significantly reduced the mean error for trainees in all three planes (Table 2). For the expert group, PSI only reduced error in the coronal plane. There was no difference between the trainees and experts using PSI (Table 1).

### Trainees with PSI vs. experts with conventional instruments

Compared to experts using conventional instruments, trainees with PSI had a significantly lower mean error in the coronal plane, and comparable mean error in the sagittal and axial planes (Table 1). There was no difference in the overall compound error between trainees using PSI and experts using conventional instrumentation.

### Measurement reliability

The mean inter-rater measurement error was 0.3° (sd 0.3°) coronal plane, 0.4° (sd 0.3°) sagittal plane, and 0.4° (sd 0.4°) axial plane. The mean intra-rater error was 0.3° (sd 0.3°), 0.4° (sd 0.3°), and 0.5° (sd 0.4°) respectively. Inter-rater (3,1) and intra-rater (1,1) intra-class correlation coefficients in all three planes indicated almost perfect agreement using the Landis and Koch criteria [18].

## Discussion

This study addressed one of the factors in the low uptake of UKA surgery, despite its many advantages to patients. Given the high degree of technical expertise required, we set out to determine whether assistive technology, in the form of low-cost 3D printed instruments, has the potential to improve surgical accuracy in low-volume surgeons.

Unsurprisingly, this study shows that overall, using conventional instruments, expert surgeons are significantly more accurate than trainee surgeons at performing the saw cuts necessary to position a medial UKA tibial component according to a pre-operative plan. However, PSI immediately allowed the same trainee surgeons, who had not previously performed a UKA, to achieve the same level of overall accuracy as the expert surgeons. Furthermore, for coronal alignment PSI actually made the trainees, and experts, significantly more accurate than the experts using conventional instruments. Despite this, the PSI cutting guide had no impact on the overall accuracy of the expert surgeons.

The largest error for the expert group using conventional instruments was in the coronal plane, with no statistical difference between the experts and trainees, and significantly lower errors for both the trainees and experts using PSI. Indeed, all four conventional expert saw cuts resulted in more varus angulation than planned (2°–5°). Although this represents a failure to reproduce the pre-operative plan of a tibial component orthogonal to the tibial mechanical axis, it might reflect a subconscious desire to recreate the natural joint line obliquity, which is associated with a lower risk of UKA revision [19, 20]. This might have been exacerbated because the sawbone had a medial proximal tibial angle (MPTA) of 83°, which is outside the normal range [21].

Also interesting was the larger than expected, although not statistically significant, sagittal plane error for the expert group using PSI. This was due to a single large error of 6° by a surgeon unfamiliar with PSI, whose focus on fitting the guide to the bone meant that their normal routine of confirming extra-medullary alignment guide parallelity with the tibial shaft was overlooked. The same error befell the two outliers in the trainee PSI group, and is probably an example of inattentional blindness due to a focus on the new technology. This underlines the importance of simulation training before any technology is used in a clinical setting [22]. The latest Embody PSI design (Embody, London, UK) addresses the risk of sagittal plane error by incorporating a novel patient-specific ankle guide which fixes the posterior slope as per the pre-operative plan.

According to the manufacturer’s guidelines on acceptable implant position in the sagittal (± 5°) and coronal planes (± 5°), for the trainees PSI reduced the number of outliers from 16 to 1 [17]. For the experts, the single large error in posterior slope with PSI meant that the number of outliers increased from zero to one with PSI. These results are comparable to the three outliers reported by Kerens et al. [23] in their first 30 cases using Zimmer Biomet’s commercially available MRI-based Signature® PSI (Warsaw, Indiana).

Two randomised controlled trials (RCTs) concluded that, using 2D radiographs, that MRI-based PSI for UKA does not improve implant positioning compared to conventional instrumentation [14, 24]. However, these two RCTs were conducted by expert UKA surgeons in high-volume centres, so an alternative interpretation is that PSI allows surgeons to replicate expert results. Two previous studies have examined the role of PSI for inexperienced surgeons [25, 26]. A small sawbone study of 16 trainee surgeons, who performed lateral UKA using CT-based PSI and conventional instruments, found no difference in accuracy of implant alignment in the coronal, sagittal, or axial planes between the techniques [25]. However, a recently published clinical trial comparing 25 medial UKA performed using MRI-based PSI by a surgeon with no prior UKA experience, and 25 performed using conventional instrumentation by a surgeon ‘with wide experience’ of UKA, found no difference in tibial component alignment, patient reported outcome scores, or 2-year survival rates [26]. It should be noted that only coronal plane positioning was specified pre-operatively and unfortunately no detail was given regarding the training received by the one inexperienced UKA surgeon. Nonetheless, the results are encouraging and consistent with the findings in our study.

Other factors, such as patient selection, might contribute to the caseload effect on UKA revision rates. However, it is known that UKAs performed by low-volume surgeons are more likely to be revised for aseptic loosening and unexplained pain [9]. Evidence from previous studies suggest that tibial component positioning is an important factor in both these factors. In a retrospective review of 559 medial UKAs by Chatellard et al. [13], tibial components orientated more than 3° from the native joint line in the coronal plane were associated with decreased prosthesis survival. However, a finite element (FE) model analysis by Innocenti et al. predicted higher stresses in the underlying cancellous bone when the tibial implant was positioned outside of 0°–3° varus in the coronal plane [27]. As well as increasing the risk of aseptic loosening, higher bone stresses increase the risk of revision surgery due to pain [28]. In the sagittal plane, Chatellard et al.’s study also identified that a posterior slope greater than 5°, or a change in the native tibial slope of more than 2°, increased the risk of implant failure [13]. This is supported by biomechanical studies, with a significant increase in underlying bone strain with posterior slopes greater than 5° [29, 30]. In our study, this level of accuracy was delivered using PSI, with mean absolute error in tibial component positioning for the trainees being significantly less than 3° in the coronal and sagittal planes (*p* < 0.0001 for both). Yet, with conventional instrumentation, mean trainee error was not less than 3° in the coronal plane (*p* = 0.3382) and significantly more than 3° in the sagittal plane (*p* = 0.0259).

The cost-effectiveness of assistive technology is relevant to this study. A Markov model analysis of robotic-assisted UKA calculated an incremental cost of $150,000 per quality-adjusted life-year (QUALY) with a case volume of 32 per annum [31]. For lower-volume surgeons, who arguably have the most to gain from assistive technology, the price per QUALY would be even higher. Although PSI is undoubtedly cheaper than robotic technology, there are no published cost-effectiveness studies of PSI for UKA. In TKA, the value of PSI remains unclear; two studies have concluded that associated improvements in operating room efficiency offset any additional costs [32, 33], whilst a study by Barrack et al. [34] reached a different conclusion. However, these studies are based on the assumption that PSI does not improve component alignment in TKA, which our sawbone study suggests might not be the case for low-volume UKA surgeons.

Our study has some limitations. The results only apply to the two designs of guide tested, and therefore cannot be generalised. Oxford Phase III instruments (Zimmer Biomet, Bridgend, UK) instruments were used in this study. A newer instrumentation set (Oxford Microplasty) has been developed by the same manufacturer, but uses a similar instrument to guide tibial resection in the coronal, sagittal, and axial planes. Indeed, a recent study by Walker et al. [35] comparing their first 100 cases using the Phase III instruments, with their first 100 cases using the Microplasty instruments, found no difference in the accuracy of tibial component positioning. Trainees without prior experience of UKA were tested, and it is unknown whether their results are representative of low-volume UKA surgeons. Albeit, the most common mean UKA caseload in the UK is one per year, and so arguably these surgeons are not much more familiar with the procedure than a trainee. It is also important to recognise that the results probably represent a best case scenario for both instruments, because, in vivo, the visualisation of key landmarks through a mini-arthrotomy is more challenging, and soft tissue makes PSI positioning more difficult. It was assumed that the tibial implant sits perfectly on the cut bone surface, and so any potential variability between expert and trainee cementation technique was not considered. However, this would not be a factor when using the newer cementless Oxford implants (Zimmer Biomet, Bridgend, UK), which have been shown in an independent series to be a safe alternative to the cemented version [36]. The decision to examine tibial component alignment, but not resection depth, was a conscious one given that resection depth is an intra-operative decision based on soft tissue tension.

## Conclusion

Higher UKA revision rates in inexperienced or low-volume surgeons has led to the suggestion that the procedure should only be carried out in expert centres [9–11]. However, with almost 50% of patients suitable for UKA, this may not be practical [37]. Assistive technology to improve component positioning is one solution and, whilst robotics are unlikely to be economically viable in a low-volume practice, PSI appears ideally placed to help [31, 32].

This study examined the difference in UKA tibial saw cuts between expert and non-expert surgeons. It demonstrates that in a sawbone model, the novel PSI tested allows inexperienced surgeons to achieve the same level of accuracy as expert surgeons. The next step is to ascertain if these results can be replicated in a clinical trial. Whether these improvements translate to improved patient outcomes and lower revision rates is, in part, likely to depend on the quality of the pre-operative plan, and the use of expert trained planning algorithms might be necessary.
